# Repeated treatment of recurrent uncomplicated *Plasmodium falciparum *malaria in Senegal with fixed-dose artesunate plus amodiaquine versus fixed-dose artemether plus lumefantrine: a randomized, open-label trial

**DOI:** 10.1186/1475-2875-10-237

**Published:** 2011-08-12

**Authors:** Jean-Louis A Ndiaye, Babacar Faye, Ali Gueye, Roger Tine, Daouda Ndiaye, Corinne Tchania, Ibrahima Ndiaye, Aichatou Barry, Badara Cissé, Valérie Lameyre, Oumar Gaye

**Affiliations:** 1 Department of Medical Parasitology, Medical Faculty, Université Cheikh Anta Diop, Dakar, Senegal; 2 District of Ndoffane, Ministry of Health, Senegal; 3 London School of Hygiene and Tropical Medicine, UK; 4 Access to Medicines Department, Sanofi Aventis Group, Paris, France

## Abstract

**Background:**

The use of artemisinin-based combination therapy (ACT) is currently recommended for treating uncomplicated malaria. The objective was to assess the efficacy and safety of repeated administrations of two fixed-dose presentations of ACT - artesunate plus amodiaquine (ASAQ) and artemether-lumefantrine (AL) - in subsequent episodes of *Plasmodium falciparum *malaria.

**Methods:**

A randomized comparative study was conducted in a rural community of central Senegal from August 2007 to January 2009. Children and adults with uncomplicated *P. falciparum *malaria were randomized to receive open-label ASAQ once daily or AL twice daily for three days. Drug doses were given according to body weight. Treatments for first episodes were supervised. For subsequent episodes, only the first intake of study drug was supervised. ECGs and audiograms were performed in patients ≥12 years of age. Primary outcome was adequate clinical and parasitological response rate (ACPR) after polymerase chain reaction (PCR) correction on day 28 for the first episode.

**Results:**

A total of 366 patients were enrolled in the two groups (ASAQ 184, AL 182) and followed up during two malaria transmission seasons. In the intent-to-treat population, PCR-corrected ACPRs at day 28 for the first episode were 98.4% and 96.2%, respectively, in the ASAQ and AL groups. For the per-protocol population (ASAQ 183, AL 182), PCR-corrected ACPRs at day 28 for the first episode were 98.9% and 96.7%, respectively. A 100% ACPR rate was obtained at day 28 in the 60 and four patients, respectively, who experienced second and third episodes. Treatment-related adverse events were reported in 11.7% of the patients, without significant differences between the two groups. A better improvement of haemoglobin at day 28 was noted in the ASAQ versus the AL group (12.2 versus 11.8 g/dL; p = 0.03). No sign of ototoxicity was demonstrated. A prolongation of the QTc interval was observed in both groups during treatment with no clinical consequence.

**Conclusions:**

Study results confirmed the satisfactory efficacy and safety profile of ASAQ and AL. Moreover, in patients who were treated at least twice, repeated administration of ASAQ or AL did not identify any major safety issues.

**Trial registration:**

ClinicalTrials.gov identifier NCT00540410.

## Background

Malaria is a leading cause of morbidity and mortality, particularly in children, in sub-Saharan Africa, the infection being due to *Plasmodium falciparum *in 98% of cases [1]. It is estimated that in 2008, among the children living in Africa, there were 247 million cases of malaria. Nearly one million of these children died of the disease, making malaria responsible for one-fifth of all childhood deaths in Africa. In Senegal alone, two million cases of malaria were recorded in 2005, and 2000 deaths were attributed to the disease [2].

The World Health Organization (WHO) first recognized the benefit of oral artemisinin-based combination therapy (ACT) compared with oral monotherapy for the treatment of uncomplicated *P. falciparum *malaria in 2001 [3], and the 2006 guidelines recommended the use of ACT [4]. This recommendation has been reiterated in the second edition of its guidelines published in 2010 [5]. The rationale for this recommendation is that the artemisinin derivative very rapidly reduces the parasite biomass, thus enabling lasting exposure of the few remaining parasites to a high concentration of the other agent in the combination [6]. The artemisinin derivative may also prevent transmission of *P. falciparum *and thus limit the spread of resistance [7]. The use of ACT was instigated in Senegal in 2006 as part of the National Malaria Control Programme (NMCP), which involved both prevention and prompt treatment. This policy decision was supported by the finding that ASAQ used over a six-year period remained highly effective in a region of Senegal where chloroquine resistance exceeded 60% [8]. Following this decision, a large reduction in the number of confirmed malaria cases was seen, and this approach is considered to be an important contributory factor in the 30% reduction in all-cause mortality that occurred between 2005 and 2009 in Senegal among children under five years of age [2].

In clinical trials conducted in Africa, oral once-daily artesunate plus once-daily amodiaquine (ASAQ) given for three days has been shown to be as effective and as well tolerated as comparators in the treatment of uncomplicated *P. falciparum *malaria [9-14]. The use of a fixed-dose combination (FDC) helps eliminate the risk of only one of the two drugs being taken, which may be an especially important consideration in children [15]. The benefits of a FDC therapy have been recognized by the WHO, being considered to be preferable to blister co-packaged or loose tablet combinations for the treatment of malaria [5]. The Artesunate Amodiaquine Winthrop^® ^FDC (Coarsucam^®^, Sanofi Aventis) was developed in 2007 by Sanofi-Aventis' Impact Malaria in partnership with the Drugs for Neglected Disease initiative, a non-profit-making drug research and development organization. ASAQ and artemether-lumefantrine (AL) (Coartem^®^, Novartis) are two co-formulated ACTs to be granted "prequalified" status by the WHO, with the aim of ensuring that active and effective anti-malarial treatment reaches those people in need. In Senegal, the NMCP has recommended the use of either ASAQ or AL as first-line treatment since 2006, when it became available in the public sector. Since then, more than 1.5 million treatments have been administered.

People living in areas where the disease is endemic often experience more than one malaria attack in a single season, depending on the local epidemiological profile. The aim of this randomized, open-label study was to compare the clinical and parasitological efficacy, in addition to clinical safety, laboratory profile, QTc and auditory safety, of repeated administration of ASAQ FDC versus AL FDC for the treatment of recurrent episodes of uncomplicated *P. falciparum *malaria in both adults and children.

## Methods

### Study design

The study was conducted at a single centre in Senegal over two periods of malaria transmission. The study site was the rural community of Keur Soce, which is 200 km south-east of Dakar. Residents in this community had previously shown a strong commitment to the treatment of malaria and recognized the role of the community health workers who visited the patients weekly. On day 0, patients presenting with symptoms of uncomplicated *P. falciparum *were randomized to open-label oral treatment with either once-daily ASAQ FDC or twice-daily AL FDC taken on three consecutive days (days 0-2) under the supervision of a dedicated nurse; clinical and parasitological investigators were not aware of the type of allocated ACT. ASAQ was shipped to Senegal by Sanofi Aventis, and AL was purchased from a wholesaler in Dakar. The once-daily dose of ASAQ was adjusted according to patient's body weight: 5.0-8.9 kg, one 25.0/67.5-mg tablet per day; 9.0-17.9 kg, one 50/135-mg tablet; 18.0-35.9 kg, one 100/270-mg tablet; and ≥36 kg, two 100/270-mg tablets. The number of 20/120-mg AL FDC tablets taken daily was adjusted according to body weight: 5.0-14.9 kg, one tablet twice daily; 15.0-24.9 kg, two tablets twice daily; 25.0-34.9 kg, three tablets twice daily; and ≥35 kg, four tablets twice daily. Tablets were taken with a small volume of potable water. If the patient vomited within 30 minutes of tablet intake, the dose was repeated. In the event of a second vomiting episode, the patient was withdrawn and received quinine in compliance with the directives of the NMCP. In addition, paracetamol 60 mg (maximum daily dose 3 g) was permitted for the treatment of fever. Patients were evaluated during treatment (days 0-2), on day 3 and at follow-up on days 7, 14 and 28. Patients failing treatment within the 14 days of the initiation of study-drug treatment received quinine in accordance with the NMCP directive. Residents of the villages within the community were regularly visited by the community health workers. Patients with fever and sickness were asked to attend the clinic. Any patient identified as presenting with a recurrent episode occurring more than 14 days after the first episode received the same treatment as taken for the first episode (but with only the first drug intake being supervised). A recommendation to take food immediately after the unobserved ACT intake was given by the nurse in charge of treatment allocation. In the event of vomiting within the 30 minutes of unsupervised medication intake, the patient was advised to return to the centre in order to be re-administered the same dose. Patients were evaluated at days 0, 3, 7, 14 and 28. The protocol complied with recommendations of the 18th World Health Congress (Helsinki, 1964), and all applicable amendments, conformed to the laws and regulations of Senegal and received local Ethics Committee approval. The study obtained the ethical approval from the Conseil National de Recherche en Santé (National Council for Health Research) of Senegal on 7 August 2007.

### Patients

Finger-prick blood samples were obtained using a vaccinostyle and were prepared and stained with May-Grünwald-Giemsa. Thick smears were used to determine parasite density, and thin smears were used to characterize the parasite species. The inclusion criteria were: *P. falciparum *infection with a blood parasite density >1000 asexual forms/μL on day 0 of first episode of malaria; body weight ≥5 kg; axillary temperature ≥37.5°C (measured using an electronic thermometer on day 0 or history of fever within the previous 24 hours (not required if treatment failure according to the WHO classification [5] occurred >14 days after a previous episode); and provision of written informed consent of adult patients or that of a parent or guardian for subjects <18 years of age. The presence of any of the following excluded patients from the study: pregnancy confirmed by a positive urine test; history of hepatic and/or haematological impairment during treatment with amodiaquine; family history of congenital QTc prolongation or sudden death, or any clinical condition known to prolong the QTc interval (e.g., cardiac arrhythmia with significant bradycardia or severe cardiac failure); allergy to any component of the study drugs; presence of one or more serious or clinical danger signs of malaria; receipt of medication metabolized by or inhibiting CYP2D6; receipt of medication known to prolong the QTc interval; electrolyte imbalances; and/or receipt of ASAQ or AL at an appropriate dosage ≤14 days before inclusion in this study.

### PCR analysis

DNA was extracted from blood collected on 3MM^® ^Whatman filter paper by the methanol method [16]. Extracted DNA from each sample was used immediately for PCR by a two-step amplification scheme, with the product of the outer PCR being used as the template for the nested-PCR for both *msp1 *and *msp2*. For *msp1*, the primers used in the first amplification were conserved among all isolates. Allelic family-specific primers used in the second amplification reaction for block2 of *msp1 *corresponded to *msp1 *types MAD20, KI and RO33. PCR amplifications of *msp2 *genes were performed as described by Foley *et al *[17]. The *msp2 *nested-PCR product was subjected to site-specific restriction digestion using *Haemophilus influenza *1 enzyme to distinguish between type FC27 and 3D7 alleles. Electrophoresis was performed on the PCR products, using 100 base pair ladders to estimate the sizes of the DNA fragments.

### Study endpoints

The primary efficacy endpoint was adequate clinical and parasitological response (ACPR), as defined by WHO [18], of ASAQ compared with AL after polymerase chain reaction (PCR) correction at day 28 of the first episode. The secondary efficacy endpoints were: ACPR efficacy at day 14 of first episode; time to parasite clearance after the first episode; time to fever resolution after first episode; ACPR at days 14 and 28 of second and any subsequent episodes; proportion of apyretic patients at day 3 of second and any subsequent episodes; proportion of gametocytes carriers and proportion of patients free from parasite at day 3 of second and any subsequent episodes. The nature and intensity of all adverse events were assessed by the investigator at each visit until day 28 by questioning the patient/parent/guardian. All events were recorded as treatment-emergent if they occurred between day 0 and day 28. Some adverse events were defined as of special interest as described in the study protocol, together with the date of onset. An adverse event of special interest is defined as one that requires appropriate continuous monitoring and could necessitate additional examinations for it to be characterized and understood. Clinical laboratory tests and vital signs after each episode were also monitored. Venipuncture samples collected on days 0, 7 and 28 were analysed for haematological parameters (haemoglobin and platelet, neutrophil and leucocyte counts) and biochemical parameters (blood glucose, blood creatinine, total bilirubin and alanine aminotransferase). In addition, QTc interval was calculated using the Fridericia formula from an ECG (Philips Trim III) and was assessed on day 0 before dosing and on day 3 of the first episode in patients aged ≥12 years. Auditory evaluation was performed by determining the difference (in decibels) observed between the hearing threshold obtained for each ear and octave range using an AUDIOSCAN^® ^(Essilor International; Vincennes, France) high-resolution automatic Békésy audiometry method, involving level frequency sweeps and between 5 and 16 kHz, performed in subjects without a history of otological pathology and aged ≥12 years after the first and any subsequent episode on day 0 before dosing and on day 3, and was planned to be repeated on day 28 if any abnormality had been detected on day 3. Both QTc prolongation and audiometric assessment were performed by independent investigators. Treatment compliance was determined by tablet count of returned medication after the second and any subsequent episodes. All adverse events were coded using the Medical Dictionary for Regulatory Activities, and severity of clinical laboratory abnormalities was graded according to the Division of Microbiology and Infectious Disease Toxicity Tables (May 2001).

### Statistical methods

Non-inferiority of ASAQ compared with AL was tested by calculating the 95% confidence interval (CI) of the difference observed in the success rates between both treatment groups, with a 2.5% (one-sided) significance level (non-inferiority delta of 5%). It was established that a total of 174 patients would need to be enrolled based on a previous AL study that reported a treatment failure rate of 2.7% [19]. Allowing for 15% incidence of premature withdrawals, the total number of patients required would be 200 per treatment group.

Statistical analyses were performed using SAS^® ^software package (version 8.2: SAS Institute Inc., Cary, NC, USA). Analysis of the primary efficacy endpoint was conducted in the intent-to-treat population (i.e., all randomized patients having received at least one treatment dose). Analysis of the efficacy was also performed in the per-protocol population. The non-inferiority of ASAQ versus AL was determined by calculating the 95% CI of the difference observed in the PCR-corrected ACPR between the two treatment groups, with a 2.5% (one-sided) significance level (non-inferiority difference of 5%). Time to the next episode was analysed using Kaplan-Meier survival plots. The statistical significance of the mean difference between treatment groups was determined using Student t-test, or Wilcoxon rank test when distribution was not normal. Percentage responses were analysed by the chi^2 ^test or Fisher's non-parametric test when the number of patients was less than five. Wilcoxon rank test was used to compare total bilirubin and alanine aminotransferase levels between treatment groups during the first episode.

## Results

### Patient characteristics

**Figure 1:**
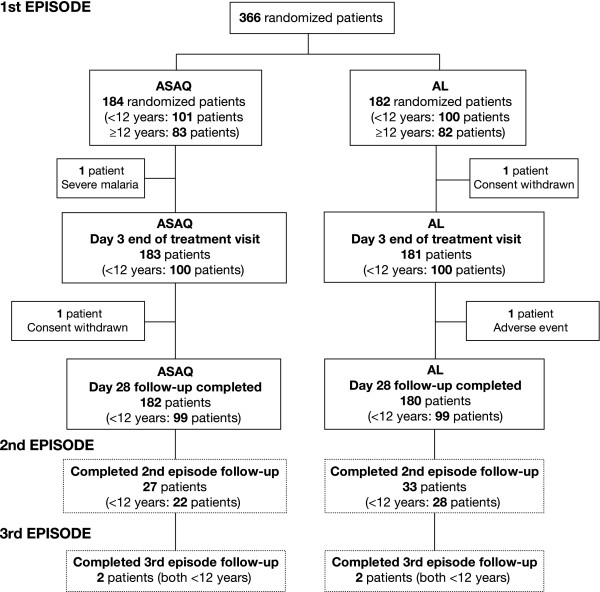
**Disposition of patients according to the number of malarial episodes experienced in a randomized open-label study: comparing treatment with fixed-dose combinations of artesunate plus amodiaquine (ASAQ) and artemether plus lumefantrine (AL)**.

A total of 839 patients were screened. Reasons for non-enrolment, in addition to failure to meet the inclusion criteria and/or the presence of an exclusion criterion, were unwillingness to commit to two years' follow-up and logistical problems in visiting the health centre. A consort flow diagram is shown in Figure 1, which summarizes the withdrawals and the number of patients experiencing second and third episodes in the 366 randomized patients. Because of the vigilance of the community health workers, no patient was lost to follow-up. The baseline characteristics of the 366 patients enrolled and randomized to either ASAQ FDC (n = 184) or AL FDC (n = 182) were comparable (Table 1). No differences in vital signs, malaria signs and symptoms (pain, headache, nausea, chills, perspiration, weakness, anorexia, jaundice, dizziness, dehydration and splenomegaly), or axillary temperatures were apparent in the two treatment groups. The predominant symptoms were mild weakness and anorexia.

**Table 1:** Patient characteristics of the intent-to-treat population^a^

Characteristic	ASAQ (n = 184)	AL (n = 182)	p-value
Sex, n (%)			
Male	102 (55.4)	98 (53.8)	0.76^b^
Age, years			
Mean ± SD	11.94 ± 10.52	11.75 ± 9.17	0.67^c^
Range	0.8-65.0	1.2-60.0	
<12 years, n (%)	101 (54.9)	100 (54.9)	0.99^b^
Weight, kg			
Mean ± SD	29.55 ± 16.11	31.08 ± 17.34	0.59^c^
Range	9.0-89.0	7.6-77.2	
BMI, kg/m^2^			
Mean ± SD	14.87 ± 3.11	14.99 ± 3.22	0.58^c^
Range	10.0-29.7	9.8-27.4	
Parasite density, asexual forms/μl			
Geometric mean	32383.9 ± 37362	31713.7 ± 37253	0.716^c^
Range	1034-195764	1016-195837	
Gametocyte carriers, n (%)	7 (3.8)	2 (1.1)	0.174^d^
Blood haemoglobin, g/dL			
Mean ± SD	10.9 ± 2.3	10.8 ± 2.5	0.85^e^
Range	5.8-15.8	5.5-18.2	

^a^All randomized patients receiving at least one dose of study drug (patients vomiting twice after receipt of first dose were excluded); ^b^Chi^2 ^test; ^c^Wilcoxon rank test; ^d^Fisher's exact test; ^e^Student t-test.

### Compliance

Compliance with study-drug medication, which was taken under supervision for the treatment of the first episode, was confirmed as 100%. Compliance with treatment for the second episode was 98.3%. One patient took only one of the two tablets of AL required to be taken in the evening of day 0, but this mistake was identified by a local guide visiting the patient's village in the morning of day 1, and the need to take two AL tablets was explained again. This patient took two AL tablets twice daily for the remainder of this treatment course.

### Clinical and parasitological efficacy: primary endpoint

The PCR-corrected ACPR rate at follow-up on day 28 after the first malaria episode for the ASAQ group was 98.4% versus 96.2% for the AL group in the intent-to-treat population (Table 2). The 95% CI of the difference in the PCR-corrected ACPR rates in the intent-to-treat population for ASAQ and AL was -0.011, 0.056 in the overall population, with the non-inferiority of ASAQ versus AL being demonstrated in the overall population and in patients aged <12 and ≥12 years. The ACPR rates were comparable in the per-protocol population (Table 3), with the 95% CI of the difference in the PCR-corrected ACPRs for ASAQ and AL being (-0.008, 0.052) in the overall population. Kaplan-Meier analysis did not identify any significant difference up to day 28 between the time to treatment failure for the two treatment groups (p = 0.20).

**Table 2:** Treatment responses at day 28 after PCR correction for the first episode in the intent-to-treat patient population^a^

Treatment response	ASAQ	AL	Total
	
	Number (%)		
**Overall population**	**(n = 184)**	**(n = 182)**	**(n = 366)**
Late clinical failure	0 (0.0)	3 (1.6)	3 (0.8)
Late parasitological failure	1 (0.5)	2 (1.1)	3 (0.8)
Adequate clinical and parasitological response	181 (98.4)	175 (96.2)	356 (97.3)
Not applicable	2 (1.1)	2 (1.1)	4 (1.1)
**<12 years old**	**(n = 101)**	**(n = 100)**	**(n = 201)**
Late clinical failure	0 (0.0)	3 (3.0)	3 (1.5)
Late parasitological failure	1 (1.0)	1 (1.0)	2 (1.0)
Adequate clinical and parasitological response	98 (97.0)	95 (95.0)	293 (96.0)
Not applicable	2 (2.0)	1 (1.0)	3 (1.5)
≥**12 years old**	**(n = 83)**	**(n = 82)**	**(n = 165**)
Late parasitological failure	0 (0.0)	1 (1.2)	1 (0.6)
Adequate clinical and parasitological response	83 (100.0)	80 (97.6)	163 (98.8)
Not applicable	0 (0.0)	1 (1.2)	1 (0.6)

^a^All randomized patients receiving at least one dose of study drug (patients vomiting twice after receipt of first dose were excluded).

**Table 3:** Treatment responses at day 28 after PCR correction for the first episode in the per- protocol patient population^a^

Treatment response	ASAQ	AL	Total
	Number (%)		
**Overall population**	**(n = 183)**	**(n = 181)**	**(n = 364)**
Late clinical failure	0 (0)	3 (1.7)	3 (0.6)
Late parasitological failure	1 (0.5)	2 (1.1)	3 (0.8)
Adequate clinical and parasitological response	181 (98.9)	175 (98.7)	356 (97.8)
Not applicable	1 (0.5)	1 (0.6)	2 (0.5)
**<12 years old**	**(n = 100)**	**(n = 100)**	**(n = 200)**
Late clinical failure	0 (0.0)	3 (3.0)	3 (1.5)
Late parasitological failure	1 (1.0)	1 (1.0)	2 (1.0)
Adequate clinical and parasitological response	98 (98.0)	95 (95.0)	193 (96.5)
Not applicable	1 (1.0)	1 (1.0)	2 (1.0)
≥**12 years old**	**(n = 83)**	**(n = 82)**	**(n = 164)**
Late parasitological failure	0 (0.0)	1 (1.2)	1 (0.6)
Adequate clinical and parasitological response	83 (100.0)	80 (97.6)	163 (99.8)

^a^All patients who completed the study protocol without any major protocol deviation in treatment evaluation.

### Clinical and parasitological efficacy: secondary endpoints

Treatment response rates before PCR correction are summarized in Table 4. The calculated 95% CI of the difference of the ACPR rates before PCR correction for ASAQ and AL confirmed that the efficacy of ASAQ was non-inferior to that of AL in the intent-to-treat (-0.020, 0.065) and per-protocol (-0.022, 0.067) patient populations The ACPR rates before PCR correction at day 28 were 100% in both the intent-to-treat and per-protocol populations experiencing a second episode following treatment with either ASAQ (both populations n = 27) and AL (n = 33 and 23, respectively). For the four patients experiencing a third episode, the ACPR before PCR correction at day 28 were 100% following treatment with either ASAQ (n = 2) or AL (n = 2).

**Table 4:** Treatment responses at day 28 before PCR correction for the first episode in the intent-to-treat (ITT) and per-protocol (PP) patient populations

Treatment response	ASAQ	AL	Total
	Number (%)		
**ITT population**	**(n = 184)**	**(n = 182)**	**(n = 366)**
Late clinical failure	1 (0.5)	5 (2.7)	6 (1.6)
Late parasitological failure	4 (2.2)	4 (2.2)	8 (2.2)
Adequate clinical and parasitological response	177 (96.2)	171 (94.0)	348 (95.1)
Not applicable	2 (1.1)	2 (1.1)	4 (1.1)
**PP population**	**(n = 183)**	**(n = 181)**	**(n = 364)**
Late clinical failure	1 (0.5)	5 (2.8)	6 (1.6)
Late parasitological failure	4 (2.2)	4 (2.2)	8 (2.2)
Adequate clinical and parasitological response	177 (96.7)	171 (94.5)	348 (95.6)
Not applicable	1 (0.5)	1 (0.6)	2 (0.5)

**Figure 2:**
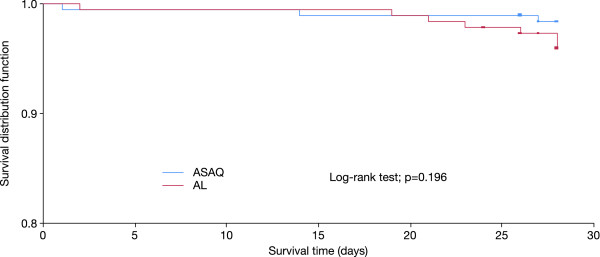
**Effects of 3 days' treatment with fixed dose combination of artesunate plus amodiaquine (ASAQ) or artemether plus lumefantrine (AL) started on day 0 of the first episode of malaria on the rate of parasite clearance**.

In the ASAQ and AL groups, 19.6% and 25.3%, respectively, received paracetamol for fever at the discretion of the physician. Fever resolution was rapid and all patients were apyretic by day 3 of the first episode. The mean time to parasite clearance was 1.7 ± 0.5 days in both the ASAQ and AL treatment groups (p = 0.10). The majority of patients (97% in both groups) were parasite-free at day 2 of the first episode, and all patients were parasite-free at day 3 (Figure 2). Although the proportion of gametocyte carriers in the ASAQ group was higher than that in the AL group at day 2 (8.2% versus 2.7%, p = 0.02), day 3 (8.2% versus 2.8%, p = 0.02) and day 7 (6.6% versus 1.1%, p = 0.01), no gametocyte carrier was detected in either treatment group at day 28.

**Figure 3:**
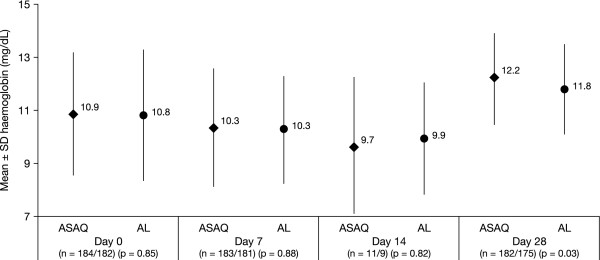
**Blood haemoglobin levels following treatment with a fixed-dose combination of artesunate plus amodiaquine (ASAQ) or artemether plus lumefantrine (AL) for the first malaria episode in the intent-to-treat population**.

Mean measured haemoglobin blood levels according to treatment groups for the first treated episode of malaria are shown in Figure 3. Haemoglobinaemia increased by 1.32 ± 2.2 and 0.94 ± 2.2 g/dL, respectively, at day 28 in the ASAQ and AL groups. The difference between the treatment groups was statistically significant (p = 0.03).

An ACPR was observed at day 28 in 60 and four patients, respectively, who presented with a second and a third episode of uncomplicated malaria, with all patients parasite-free at day 3. The mean time to first recurrence (181.3 ± 165.2 days) was not significantly different between the two treatments (p = 0.35). Of the 27 ASAQ patients experiencing a second episode, in 33% this occurred on or before day 15 after the end of the first episode, in 19% between day 16 and 41, and recurrence in remaining 46% of patients occurred 200 days or later after the first episode. The corresponding percentages for the 33 AL patients were 39%, 3% and 58%.

The ACPRs before PCR correction at day 28 were 100% in both the intent-to-treat and per-protocol populations experiencing a second episode following treatment with either ASAQ (both populations n = 27) and AL (n = 33 and 23, respectively). For the four patients experiencing a third episode, ACPR rates before PCR correction at day 28 were 100% following treatment with ASAQ (n = 2) or AL (n = 2),

### Safety

The incidence of adverse events throughout the study period is summarized in Table 5. A woman treated with AL had a positive pregnancy test at day 28. She spontaneously aborted 8 weeks after her last menstruation. There were insufficient data to establish whether or not the pregnancy and miscarriage were related to AL. The two serious adverse events (severe coma and severe convulsions), both occurring in patients aged <12 years, were not considered related to treatment with ASAQ or AL. Adverse events associated with the blood and lymphatic, gastrointestinal and nervous systems were the most common ones considered by the investigators to be treatment related, occurring at frequencies of 5.2%, 4.1% and 2.5%, respectively, throughout the study period. A summary of adverse events related to study drug treatment occurring throughout the study according to intensity is provided in Table 6. There were only four treatment-related adverse events occurring during recurrent episodes, which were mild, and the patients recovered rapidly. Treatment-related anaemia, which occurred during the first episode in five patients aged <12 years and in one patient aged ≥12 years treated with ASAQ, and in four patients aged <12 years and in four patients aged ≥12 years in the AL group, was generally mild in intensity. Recovery usually occurred with or without iron-based treatment within 3 weeks. Persistent anaemia was detected in three patients (one in ASAQ group and two in AL group) with concomitant infections or infestations. No anaemia was detected in patients treated for subsequent episodes of malaria. The severe neutropenia (<400 neutrophils/mm^3 ^in children and <750 neutrophils/mm^3 ^in adults) observed in 10 patients (four in the ASAQ group, six in the AL group) was considered to be treatment related in five patients; all were ≥12 years of age. Recovery by day 28 was observed in two patients, and neutrophil counts subsequently returned to normal in the remaining patients. Gastrointestinal events (mild, transient abdominal pain and/or vomiting) occurred in nine (4.9%) ASAQ- and seven (3.7%) AL-treated patients, and were more frequent in patients aged ≥12 years old. Transient mild sleep disturbances occurred in seven (3.8%) patients in the ASAQ group and were more common in patients aged <12 years. These disturbances were resolved within 1 or 2 days.

**Table 5:** Adverse events (AEs) occurring during the whole study period

	ASAQ (n = 184)	AL (n = 182)
Emergent AEs	60	60
Treatment-related AEs (%)^a^	32 (53.3)	21 (35.0)
AE of special interest^b ^(%)	4 (6.7)^c^	4 (6.7)^c^
Pregnancy (%)	0	1 (1.7)^c^
Serious AEs (%)	1 (1.7)	1 (1.7)
Deaths	0	0
AE intensity (%)		
Mild	45 (75.0)	44 (73.3)
Moderate	13 (21.7)	15 (25.0)
Severe	2 (3.3)	1 (1.7)
Overall study (%)		
Patients with at least one AE	47 (25.5)	47 (25.8)
Patients with at least one treatment-related AE	26 (14.1)	17 (9.3)
Patients with at least one SAE and permanent study withdrawal due to AE	1 (0.5)	1 (0.5)
**First episode (%)**		
Patients with at least one AE	43 (23.4)	41 (22.5)
Patients with at least one SAE and permanent study withdrawal due to AE	1 (0.5)	1 (0.5)
**Second episode (%)**		
Patients with at least one AE	4 (14.8)	8 (24.2)

^a^Percentage of emergent events. ^b^Neutropenia <400 neutrophils/mm^3 ^in children or <750 neutrophils/mm^3 ^in adults. ^c^Occurred during 28 days of first episode evaluation.

**Table 6:** Incidence of treatment-related adverse events occurring during the whole study^a^

Disorder type	ASAQ (n = 184)			AL (n = 182)		
	
	Number (%)					
	
	Mild	Moderate	Severe	Mild	Moderate	Severe
Blood and lymphatic	7 (3.8)	2 (1.1)	1 (0.5)	8 (4.4)	2 (1.1)	0 (0.0)
Gastrointestinal	7 (3.8)	2 (1.1)	0 (0.0)	6 (3.3)	1 (0.5)	0 (0.0)
Nervous	7 (3.8)	1 (0.5)	0 (0.0)	1 (0.5)	0 (0.0)	0 (0.0)
Skin, subcutaneous tissue	2 (1.1)	0 (0.0)	0 (0.0)	0 (0.0)	1 (0.5)	0 (0.0)
Respiratory, thoracic, mediastinal	2 (1.1)	0 (0.0)	0 (0.0)	0 (0.0)	0 (0.0)	0 (0.0)
Hepatobiliary	1 (0.5)	0 (0.0)	0 (0.0)	0 (0.0)	0 (0.0)	0 (0.0)
General	0 (0.0)	0 (0.0)	0 (0.0)	0 (0.0)	1 (0.5)	0 (0.0)

^a^Percentage of patients experiencing an event/total safety population.

No differences in haematological or renal laboratory findings were detected in the two treatment groups. Similarly, no differences in total bilirubin and serum alanine aminotransferase were recorded (Table 7).

**Table 7:** Summary of changes in total bilirubin and alanine aminotransferase during the first episode

	ASAQ	AL	p-value
**Total bilirubin mg/dL)**			
**Day 0**	**(n = 184)**	**(n = 182)**	
Mean ± SD	1.46 ± 0.99	1.50 ± 1.51	0.796
Range	0.02-5.52	0.01-17.27	
**Day 7**	**(n = 182)**	**(n = 180)**	
Mean ± SD	0.83 ± 1.45	0.80 ± 1.04	0.825
Range	0.03-19.25	0.04-13.18	
**Day 28**	**(n = 181)**	**(n = 175)**	
Mean ± SD	0.60 ± 0.35	0.80 ± 1.04	1.000
Range	0.01-2.33	0.03-2.57	
**ALT (UI/L)**			
**Day 0**	**(n = 184)**	**(n = 182)**	
Mean ± SD	29.2 ± 30.0	27.8 ± 29.8	0.505
Range	2-199	1-217	
**Day 7**	**(n = 183)**	**(n = 181)**	
Mean ± SD	22.4 ± 15.7	25.1 ± 27.4	0.877
Range	1-95	1-218	
**Day 28**	**(n = 181)**	**(n = 175)**	
Mean ± SD	20.9 ± 14.9	19.5 ± 15.9	0.053
Range	1-110	3-99	

Audiometric measurements were performed in 167 patients (74 female patients aged 19.4 ± 10.8 years, and 93 male patients aged 18.8 ± 10.7 years) during the first treated malaria episode and in 12 patients experiencing a second episode. Hearing thresholds measured on days 3 and 28 did not show any significant variation compared with that measured prior to treatment administration on day 0, with no difference between ASAQ and either dose of AL.

**Figure 4:**
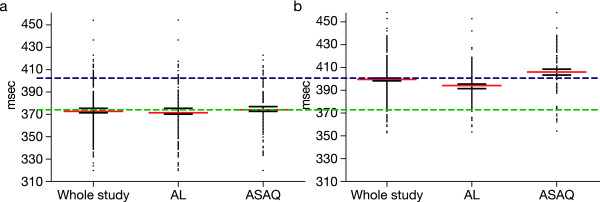
**QTcF at day 0 and day 3 after treatment with a fixed-dose combination of artesunate plus amodiaquine (ASAQ) or artemether plus lumefantrine (AL) for the first malaria episode in the intent-to-treat population**.

ECGs were recorded in 171 patients on day 0 and in 153 patients on day 3. Marked prolongations of the QTc interval (>20 ms) were observed in both treatment groups between day 0 and day 3 (Figure 4). The mean increase in the QTc interval was 22.4 ms in the AL group compared with 33.2 ms in the ASAQ group, and the difference between these treatments was statistically significant (p = 0.011). Details of ECG results are summarized in Table 8. Unfortunately, planned control at day 28 was not performed in a majority of cases due to logistical problems in transmitting the ECGs to the off-site evaluator. However, the ECG modifications at day 3 had no clinical significance or any impact; hence, the investigators did not perform an ECG control at day 28.

**Table 8:** Summary of ECG changes

	ASAQ	AL
	
	Number of episodes	
**QTcF interval at day 3**	**(n = 77)**	**(n = 77)**
>500 ms	0	0
480-499 ms	0	0
450-479 ms	3	1*
**Change in QTcF from baseline (day 3/day 0)**	**(n = 76)**	**(n = 77)**
≥60 ms	9	6
30-59 ms	30	23

* This patient presented with a QTcF > 450 ms at baseline.

## Discussion

ACT is now regarded as the first-line treatments for uncomplicated *P. falciparum *malaria in most malaria-endemic countries, as advocated by the WHO [4,5], and is gaining widespread acceptance in both the private and public sectors for the treatment of adults and children in malaria-endemic African countries. ACT, such as ASAQ, can reduce the parasite biomass rapidly and substantially, resulting in fast parasite clearance and resolution of clinical symptoms. Together with its efficacy against multidrug-resistant *P. falciparum*, its ability to reduce gametocyte carriage and to help reduce transmission of resistant alleles makes ACT an essential tool in malaria control and the fight to eliminate the disease [6]. The efficacy of ASAQ given as a FDC is supported by findings of clinical studies performed in locations throughout sub-Saharan Africa [12,13].

Pharmacovigilance, involving the detection, assessment, understanding and prevention of adverse effects or any other drug-related problems, of ACT has been advocated to establish its safety in African populations. This is especially important because such treatments may be repeatedly used in treating recurrent episodes in those geographical areas where the disease is endemic. Most notably, there have been concerns about the potential of AQ monotherapy to cause haematological and hepatic toxicity when used prophylactically [20]. Post-marketing safety usually relies on the spontaneous reporting of adverse reactions, but is often incomplete in the developing countries of sub-Saharan Africa due to an inadequate safety-monitoring infrastructure [21]. However, collaboration between academic investigators, drug companies and governments can assist in the pharmacovigilance of new anti-malarial drugs.

FDCs are important candidates for pharmacovigilance, given their potential advantages in the management of malaria. The FDC of AL was the first fixed-dose ACT to be approved, after the demonstration of its efficacy in the treatment of uncomplicated *P. falciparum *malaria when administered twice daily for three days. Subsequently, the FDC of ASAQ became available. This product only requires a three-day dosing regimen, but also offers the added convenience of once-daily dosing.

The present collaborative Phase IV study was conducted in a rural area of Senegal where malaria is endemic and highly seasonal, occurring between September and December, with an entomological inoculation rate of between 9 and 12 during that period (Faye O, unpublished data). The vector responsible for malaria transmission is *Anopheles gambiae*. In order to increase the possibility of repeat malaria episodes, the study was conducted over two periods of malaria transmission.

The present study confirmed that the once-daily dosing regimen of the ASAQ FDC did not compromise efficacy. The PCR-corrected ACPR rate - the primary efficacy endpoint - achieved with ASAQ FDC was non-inferior to that of the AL FDC administered twice daily. The resolution of fever and elimination of parasites was rapid with either treatment. Patients who are asymptomatic, however, may remain gametocyte carriers and serve as an important reservoir for further disease transmission [22]. Without eradication of gametocytes, asymptomatic infection may persist beyond the rainy season and perpetuate disease transmission into the next season [23]. The relatively small number of patients returning with a second, and particularly a third, malaria episode suggests that there may have been an effective eradication of sexual parasites in this study. Both FDCs proved equally effective in treating the second and third episodes.

Adherence to medication is fundamental to the successful treatment of malaria and is assisted by use of a FDC. Direct supervision ensures total treatment compliance by the patient, as was the case for the treatment of the first episode in this study. In day-to-day practice, however, this approach is not feasible and the health-care worker is reliant on the patient understanding and following instructions. Therefore, the simpler the regimen, the greater the likelihood is of total compliance. Although there is potential for poor adherence to dosing that involves taking more than one tablet on each occasion in the absence of supervision, there was only one patient in this study who did not take the full dose when unsupervised. However, the mistake made by this patient was quickly identified (only one dose was missed) and the problem was rectified. Compliance is likely to be poorer if a patient is not participating in a trial or is not being closely monitored. The simplicity of dosing using a FDC may also be particularly advantageous when treating children [24].

The clinical safety in terms of adverse events and clinical laboratory findings was found to be comparable for ASAQ and AL in our study, with no evidence of haematological and hepatic toxicity associated with the use of ASAQ. In our study, based on observations in nearly 400 patients, treatment-related adverse events were recorded in <12% of the patients. The incidences of these events were comparable with the ASAQ and the AL FDCs, with most events being mild or moderate in intensity. The persistent anaemia observed in three children was probably attributable to helminthiasis as prevalence of intestinal parasites in children is high in this area of Senegal. Although a relatively small proportion of patients received a second or third course of treatment for subsequent episodes, there was no evidence of any difference in adverse event reporting with repeated use of either FDC. Similarly, there was no evidence of a negative impact of either FDC on clinical laboratory findings.

An important feature of the present study is that, in addition to the monitoring of safety as routinely performed in clinical studies, the possible effects of ASAQ and AL on the impairment of hearing and the QTc interval were evaluated. Audiometric assessments did not detect any significant deleterious auditory effects in normal hearing associated with either FDC given for 3 days in adult patients. A QTc prolongation was observed in both treatment groups between day 0 and day 3. The prolongation of the QTc interval observed in patients experiencing malaria episode resulted probably from a real effect of treatments on depolarization, as well as from an interfering artefact due to fever resolution and its induced chronotropic effect, this later was greater in the ASAQ group. It should be noted that no cardiac adverse events occurred during the follow-up period. No clinical impact was observed.

One of the limitations of this study was that provision of highly effective FDC treatment in a moderate and highly seasonal transmission area, such as this study area, resulted in relatively few patients presenting with a second or third episode of malaria. The safety evaluation of repeated FDC use, therefore, is restricted in the present study, but suggests that repeated use of FDC ASAQ and AL in the clinical trial setting should present no major safety issues. Nevertheless, there is a need for more extensive systems of pharmacovigilance for FDC ACT when it is regularly used in routine clinical practice [25]. Currently, adverse events often go unreported in routine clinical practice [26,27]. Health-care workers are often unfamiliar with formal reporting procedures, and tend to consider that the costs and additional workload outweigh any benefits. Improving voluntary pharmacovigilance reporting in malaria-endemic areas of Africa requires the active participation of patients and health-care workers, and should encompass both public and private sectors. Another challenge is to ensure the reliability and validity of data generated by community health-care workers for a community-based pharmacovigilance system.

## Conclusions

Once-daily ASAQ FDC is non-inferior to twice-daily AL FDC for the treatment of uncomplicated *P. falciparum *malaria. The ACPR was unaffected by the patient's age, being equally effective in patients <12 and ≥12 years of age, and the number of malaria episodes experienced during the study. The clinical safety profiles of ASAQ FDC and AL FDC were comparable, and recurrent administration of ASAQ FDC did not result in major safety issues.

## List of abbreviations used

AL: artemether-lumefantrine; ACPR: adequate clinical and parasitological response rate; ACT: artemisinin combination therapy; ASAQ: artesunate plus amodiaquine; ECG: electrocardiogram; FDC: fixed-dose combination; ITT: intent to treat; NMCP: National Malaria Control Programme; PCR: polymerase chain reaction; PP: per protocol; WHO: World Health Organization.

## Competing interests

The authors declare that they have no competing interests.

## Authors' contributions

JLN and VL were responsible for the concept of study. JLN AG, CT, IN, RT, DN, AB, VL and OG contributed to the design of the study. OG was responsible for the overall coordination of the study. RT, DN and AB supervised the study. JLN, AB, BF, AG, CT, IN, RT and DN collected the study data. BF was responsible for the PCR analysis. JLN, BF and OG were involved in preparation of the manuscript, which was read and approved by all the authors.
